# Craniotomy

**Published:** 1880-11

**Authors:** 


CRANIOTOMY.—A professional friend enquires, "How often have you found it *necessary* to resort to craniotomy during your experience as general practitioner and teacher of obstetrics? And what would you think of a medical man, who, in a practice of less than twenty years, resorted to this dreadful operation from seventeen to twenty times?" Answer.—To the first question we would reply, we have found it necessary to sacrifice the child, by craniotomy, in but *two* instances, in an experience of nearly forty years.

To the second question, we would say, that such a party is a "prodigy," and should be well ventilated in the Journals.

